# Small RNA sequencing and bioinformatics analysis of RAW264.7-derived exosomes after Mycobacterium Bovis Bacillus Calmette-Guérin infection

**DOI:** 10.1186/s12864-022-08590-w

**Published:** 2022-05-07

**Authors:** Xuehua Zhan, Wenqi Yuan, Yueyong Zhou, Rong Ma, Zhaohui Ge

**Affiliations:** 1 Department of Orthopaedics, General Hospital of Ningxia Medical University, Yinchuan, 750004 Ningxia China; 2 Clinical Medicine School, Ningxia Medical University, Yinchuan, 750004 Ningxia China

**Keywords:** Exosomal miRNA, Tuberculosis, BCG, Macrophages, Apoptosis, RNA-seq

## Abstract

**Background:**

The mechanisms through which *Mycobacterium tuberculosis* evades immune surveillance during tuberculosis (TB) infection remain complex. Previous studies have found that Mycobacteria can manipulate the miRNAs of host cells to promote their survival during host-pathogen interactions, and most of these effects occur at the cellular miRNA level. We attempted to investigate the possible related mechanisms at the exosomal miRNA level.

**Results:**

High-throughput sequencing revealed that Bacillus Calmette-Guérin (BCG) infection could alter the composition of the macrophage exosome content, and the expression levels of miRNAs in exosomes derived from the cell culture media of macrophages showed significant differences between the BCG-infected and non-infected groups. Compared with the non-infected group, 20 exosomal miRNAs were up-regulated and 7 exosomal miRNAs were down-regulated in the infection group (*p* < 0.05), of which mmu-miR-27b-3p, mmu-miR-93-5p, mmu-miR-25-3p, mmu-miR-1198-5p, mmu-let-7c-5p and let-7a-5p were significantly up-regulated. A bioinformatic analysis indicated that these differentially expressed exosomal miRNAs were involved in multiple biological processes and pathways. The target genes of top six miRNAs in up-regulated groups were positively correlated with the regulation of apoptosis.

**Conclusions:**

The expression profile of miRNA in exosomes derived from macrophage were altered after *Mycobacterium Bovis* Bacillus Calmette-Guérin infection, and the differentially expressed miRNAs were involved in multiple biological processes and signalling pathways. The top six up-regulated miRNAs and their targeted genes were predominantly correlated with the regulation of apoptosis.

## Background

Tuberculosis (TB), mainly caused by the *Mycobacterium tuberculosis* complex (MTBC), is the uppermost catastrophic infectious disease that threatens human health and safety. Although the number of newly diagnosed TB decreased from 7.1 million to 5.8 million between 2019 and 2020, there remains 1.3 million HIV-negative individuals and an additional 214,000 HIV-positive individuals died of TB in 2020 [1].

Macrophages act a central role in the innate immunity process because these cells constitute the initial cellular niche for mycobacterial pathogenesis during infection. Through intricate pathogen recognition approaches, macrophages can orchestrate multiple signalling cascades to trigger various innate immune defences including phagocytosis, apoptosis, autophagy and inflammasome activation to eliminate Mtb [2]. Moreover, Mtb has evolved a series of intelligent strategies to hijack host cell miRNAs to subvert the abovementioned functions and achieve immune evasion [3]. Numerous emerging studies have revealed that miRNAs can manipulate host macrophage defence against Mtb infection [4, 5], but a detailed and comprehensive understanding of the mechanisms remains unclear.

MicroRNAs (miRNAs) are widely conserved small noncoding RNAs with a length of 18–24 nucleotides that display an indispensable role in many physiological and pathological aspects through the posttranslational regulation of target gene expression [6]. Exosomes are a subpopulation of extracellular vesicles with a specific plasma membrane-enclosed structure with a diameter between 30 and 150 nm that can shuttle cell-specific cargo, including RNAs, proteins, lipids and DNA, to other cells both in the vicinity or at distant sites in the body. Due to their nanoscale diameter and excellent histocompatibility, exosomes have been identified as ideal natural biological carriers that play a pivotal role in information transmission and intercellular communication [7, 8]. Exosomal miRNAs can also be transferred or transported to recipient cells and induce some biological reactions in these cells. More and more studies have indicated that exosomal miRNAs control regulate genetic expressions and different cell viability in vivo and in vitro [9–11].

The majority of previous studies focused on differential expression analysis and the functions of miRNAs at the cellular level. However, we attempted to investigate the specific mechanism of immune escape adopted by Mtb based on exosomal miRNA levels by small RNA transcriptome high-throughput sequencing and bioinformatics.

## Materials and methods

### Cell and BCG culture

RAW264.7 cells preserved in our laboratory at a low passage number were cultured in a T-75 flask (Corning, USA) with Dulbecco’s modified Eagle’s medium (DMEM; Gibco, USA) containing 1% L-glutamine, 1 mM sodium pyruvate, 10% foetal bovine serum (FBS; Gibco, USA) and 1% penicillin–streptomycin at 37 °C in an 5% CO_2_ atmosphere.

*Mycobacterium bovis* Bacillus Calmette-Guérin (BCG) St. Pasteur 1173P2 strain was frozen and stored in our laboratory. The BCG bacilli were grown in Mycobacterium complete medium (Gene-Optimal, Shanghai, China) at 37 °C, which was achieved to the logarithmic phase of growth after 10 to 12 days of culture and then aliquoted. A turbidimetric assay was conducted with a spectrophotometer (Infinite 200 PRO, TECAN, Switzerland) to measure the optical density (OD) of the bacteria at a wavelength of 600 nm. Once the OD value of the culture medium reached approximately 0.9–1.0, the BCG bacilli were harvested by centrifugation for 10 minutes at 4500 rpm and then resuspended in DMEM without antibiotics as previously described [12].

### Infection assay

The cells were planted into twelve 75 cm^2^ flasks (5 × 10^6^ cells/flask) prior to the day of infection. All the cells were classified into a non-infected group (Group A) and an infected group (Group B), and each group was analysed in triplicate. On the day of infection, the macrophage monolayers were gently washed three times with phosphate buffered saline (PBS; BI, Israel) at room temperature. The cells in Group B were stimulated with BCG at a multiplicity of infection (MOI) of 10 and incubated at 37 °C in an incubator (Thermal, USA) with 5% CO_2_ atmosphere for 4 hours, whereas PBS was added to the cells in Group A as a control. After incubation, the supernatant culture medium was replaced, and the adherent monolayers were tenderly washed with warm (37 °C) PBS (BI, Israel) three times to exclude extracellular bacteria [11]. Added fresh DMEM (Gibco, USA) with 10% exosome-depleted FBS (VivaCell Biosciences, China) and continue to culture the cells for 72 hours. All of the cell nutrient media were gathered and then centrifuged 10 minutes (3000×g) to eliminate cell debris and then passed through a 0.22-μm filter unit. At least 50 ml of the cell culture supernatants from each replicate were needed to acquire sufficient amounts of exosomes for the downstream experiments.

### Exosome characterization and exosomal RNA preparation

The Cell Culture Media Exosome Purification Kits (Norgen Biotek, Canada) were used to extract exosomes from the above-described cell culture medium in accordance with the instructions. Concisely, the required cell culture supernatants were gathered, transferred into a conical tube, and centrifuged (200×g, 15 minutes) to discard cells and debris, and the cell-free medium was then transferred to a fresh conical tube for the exosome purification. ExoC buffer and Slurry E were added separately to fresh media at different proportions according to the instructions, vortexed for 10 seconds at first and then incubated for 10 minutes at room temperature. The media were remixed and centrifuged for 2 minutes at 2000 rpm. Subsequently, the ExoR buffer (600 μl) were applied to the slurry pellet, and the pellet was incubated for a quarter at room temperature and centrifuged at 500 rpm for 2 minutes. The supernatants were then moved into a fresh Mini Filter Spin column and centrifuged at 6000 rpm for 1 minute. PBS was chosen to suspend the sedimentation and preserved it at − 20 °C. Transmission electron microscopy (TEM) (Hitachi, HT-7700, Japan) and nano flow cytometry (N30E, NanoFCM, Xiamen, China) were used to characterize the purified exosomes. Total exosomal RNA isolation and extraction were performed using an exosomal RNA isolation kit (Norgen Biotek, Canada) according to the instructions. The RNA concentration and purity were detected by Agilent 2100 Bioanalyzer (Agilent Technologies, USA).

### Small RNA library construction and miRNA sequencing

The construction and sequencing of Small RNA library comply with the standard operating program provided by Illumina. A TruSeq Small RNA Sample Prep Kit (Illumina, San Diego, CA, USA) was utilized to prepare the library. After that, an Illumina HiSeq 2500 instrument was chosen to sequence the constructed library. An in-house program, ACGT101-miR (LC Sciences, Houston, TX, USA) was applied to subject the raw reads and to delete junk sequences, adapter dimers, low complexity reads and duplicates. Finally, 18 ~ 26 nucleotides distinctive sequences were mapped to precursors of specific-species in miRbase 22.0 using the Basic Local Alignment Search Tool (BLAST) to discriminate against known and novel miRNAs derived from 3p and 5p.

### Analysis of the differential expression of miRNAs

The differential expression of miRNA was analysed by the Student ‘s *t* test. Based on standardized deep sequencing, the differential expression genes were screened by *p* ≤ 0.05. Differential miRNA cluster analysis was selected to determine the cluster pattern of miRNA regulation under diverse experimental conditions. According to the statistical similarity of the miRNA expression profiles of the samples, miRNA cluster analysis was applied to intuitively display the distribution of miRNAs in different samples using the log10-transformed normalized values in the heatmap. An advanced volcano map was plotted to manifest the overall distribution and expression levels of these miRNAs.

### Prediction of the target genes

Prediction of the target genes of differentially expressed miRNAs was accomplished by TargetScan (v5.0) and Miranda (v3.3a). The overlap between the data predicted by the two algorithms was obtained. Targeted genes with a context score less than 50 obtained with the TargetScan algorithm were filtered, and those with a maximum free energy (maximal energy) greater than − 10 obtained with the Miranda algorithm were removed. The intersections between these two software programs were considered the ultimate target genes and their Gene Ontology (GO) and Kyoto Encyclopedia of Genes and Genomes (KEGG) information of these miRNAs was annotated.

### Target genes enrichment

The enrichment analysis mainly consisted of two evaluations: GO and KEGG pathway functional annotation. First, count the number of genes corresponding to each function or pathway annotation for all selected miRNAs, and then perform hypergeometric test to determine the number of genes corresponding to different terms in the annotation library. The threshold was defined as *p* value ≤0.05, and the genes satisfying this condition were considered as dominant and endogenous genes. The GO and KEGG functional enrichment analysis of the screened target genes were accomplished by using the OmicStudio tools (https://www.omicstudio.cn/too).

### Visualization of the KEGG analysis results

The target genes which involved in cell growth and death regulation were selected and used for the subsequent data visualization analysis with Metascape (https://metascape.org). Based on the membership similarities, those *p* value less than 0.01, enrichment factor more than 1.5 and a minimum count of 3 terms were gathered and classified into clusters. In addition, a protein–protein interaction (PPI) enrichment analysis was accomplished using the following databases: STRING [13], BioGRID [14], OmniPath [15], and InWeb_IM [15]. The obtained network is composed of proteins subsets that physically interacted with at least one other member on the list. The Molecular Complex Detection (MCODE) algorithm is used to classify dense network elements if the network contains 3 ~ 500 proteins [16]. Network visualization was performed using Cytoscape (v3.1.2) [17].

### Verification of exosomal miRNAs by qRT-PCR

The experimental grouping, cell culture and BCG infection procedures were the same as those previously described. Each group consisted of three biological replicates, the exosomes were isolated by ultracentrifugation (UC), and the total RNA was extracted from the exosomes. A ReverTra Ace qPCR RT Kit (TOYOBO, Japan) was used for the cDNA reverse transcription according to the instructions provided by the manufacturer. A stem–loop method was selected for the first-strand cDNA synthesis, and the primer sequences are presented in Table 1. Quantitative real-time polymerase chain reaction (qRT–PCR) was completed using a LightCycler 96 instrument (Roche, Switzerland) with SYBR Green Real-Time PCR Master Mix (TOYOBO, Japan). A relative quantitative analysis of the miRNAs was carried out by 2^-ΔΔCT^ method, and standardization was performed with respect to U6 as the reference gene. Three biological replicates of each group were included, and each sample was analysed in three independent repeated trials.

**Table 1:** List of mmu-miRNA primer sequences

No.	mmu-miRNA	Primer sequence (5′ to 3′)
**1**	mmu-let-7a-5p	RT: CTCAACTGGTGTCGTGGAGTCGGCAATTCAGTTGAGAACTATAC F: ACACTCCAGCTGGGTGAGGTAGTAGGTTGT
**2**	mmu-let-7c-5p	RT: CTCAACTGGTGTCGTGGAGTCGGCAATTCAGTTGAGAACCATAC F: ACACTCCAGCTGGGTGAGGTAGTAGGTTGT
**3**	mmu-miR-25-3p	RT: CTCAACTGGTGTCGTGGAGTCGGCAATTCAGTTGAGTCAGACCG F: ACACTCCAGCTGGGCATTGCACTTGTCTCG
**4**	mmu-miR-27b-3p	RT: CTCAACTGGTGTCGTGGAGTCGGCAATTCAGTTGAGGCAGAACT F: ACACTCCAGCTGGGTTCACAGTGGCTAAG
**5**	mmu-miR-30a-3p	RT: CTCAACTGGTGTCGTGGAGTCGGCAATTCAGTTGAGGCTGCAAA F: ACACTCCAGCTGGGCTTTCAGTCGGATGTT
**6**	mmu-miR-98-5p	RT: CTCAACTGGTGTCGTGGAGTCGGCAATTCAGTTGAGAACAATAC F: ACACTCCAGCTGGGTGAGGTAGTAAGTTGT
**7**	mmu-miR-194-5p	RT: CTCAACTGGTGTCGTGGAGTCGGCAATTCAGTTGAGTCCACATG F: ACACTCCAGCTGGGTGTAACAGCAACTCCA
**8**	mmu-miR-5110	RT: CTCAACTGGTGTCGTGGAGTCGGCAATTCAGTTGAGAATTCCAC F: ACACTCCAGCTGGGGGAGGAGGTAGAGGGTGGT
**9**	mmu-U6	RT: CGAATTTGCGTGTCATCCTTG F: CTCGCTTCGGCAGCACATATAC

### Statistical analyses

The statistical analysis was performed using GraphPad Prism 8.4 independent sample *t* - test. Statistical significance was assumed at *p*<0.05.

## Results

### Exosome characterization and exosomal RNA preparation

The supernatants from the culture media from the two groups were collected and used for the exosome isolation. The exosome morphology was evaluated by TEM. NanoFCM was used for the detection of exosome transmembrane proteins (CD9 and CD81) and to determine the particle concentration and size distribution.

**Fig. 1:**
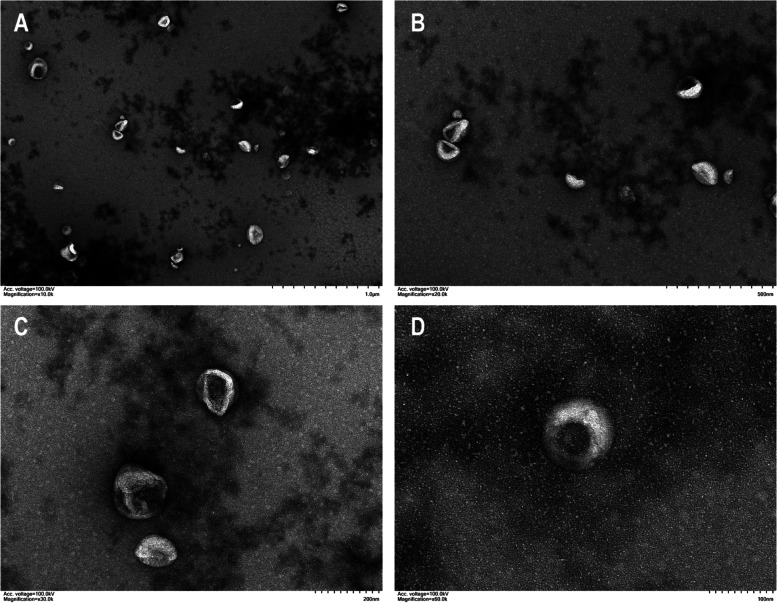
Characterization of exosomes by transmission electron microscopy (TEM). The exosome morphology was scanned by TEM. The scale bars represent 1 μm (**A**), 500 nm (**B**), 200 nm (**C**), and 100 nm (**D**)

**Fig. 2:**
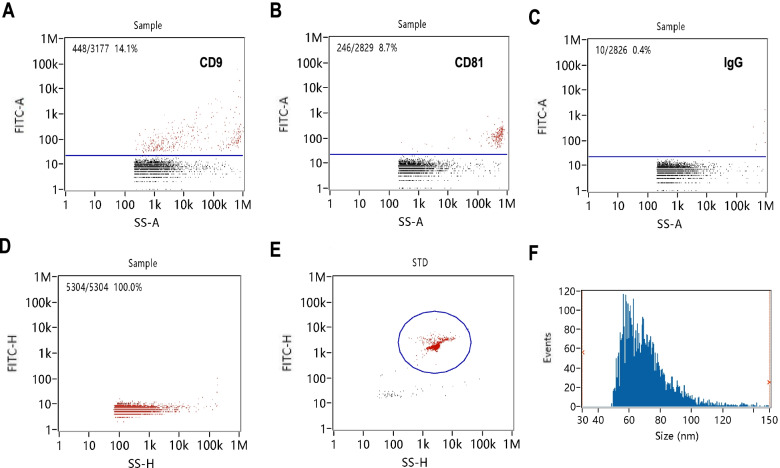
Characterization of exosomes by NanoFCM. The exosome transmembrane proteins CD9 and CD81 and isotype control FITC-IgG were detected and quantified by NanoFCM (**A**-**C**). The particle concentration was analysed by NanoFCM (**D**, **E**). The size distribution was analysed by NanoFCM (**F**)

As shown in Fig. 1, the typical morphology of exosomes can be observed by TEM; the exosome transmembrane proteins CD9 and CD91 showed positive expression in 14.1 and 8.7% of the exosomes, respectively; the average concentration was 7.45 × 10^9^ particles/ml, and the particle size was mainly distributed in the 60–80-nm range (Fig. 2). The concentration of exosomal RNA extracted from the exosomes in each sample varied from 0.0021 to 0.0039 μg/μl. Quality control and an RIN evaluation were performed, and the obtained values indicated that the samples met the standards for the subsequent sequencing analysis.

### Analysis of the differential expression of miRNAs

**Fig. 3:**
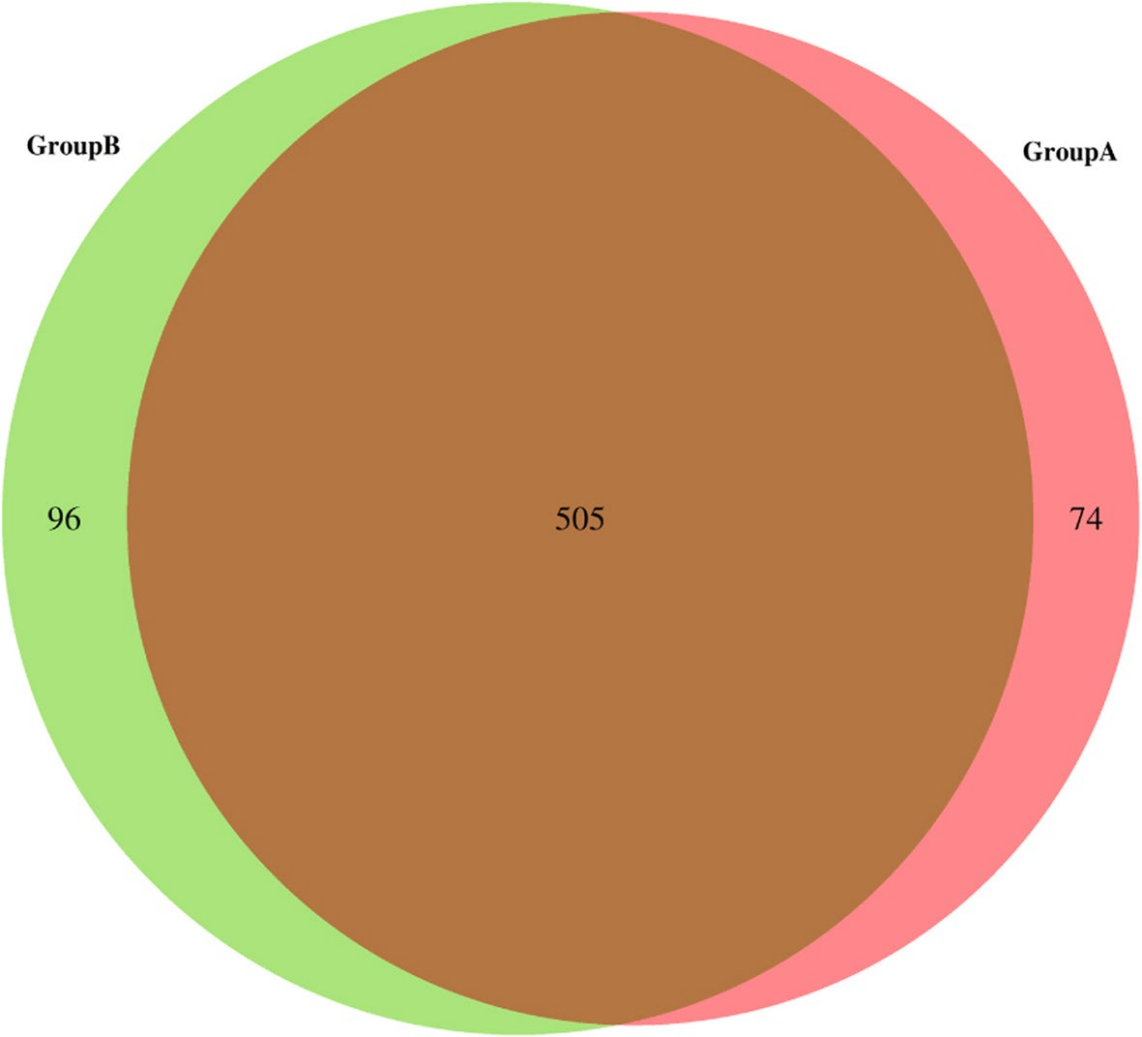
MiRNAs showing differential expression between the following two groups: Group A (noninfection group) and Group B (infection group)

**Fig. 4:**
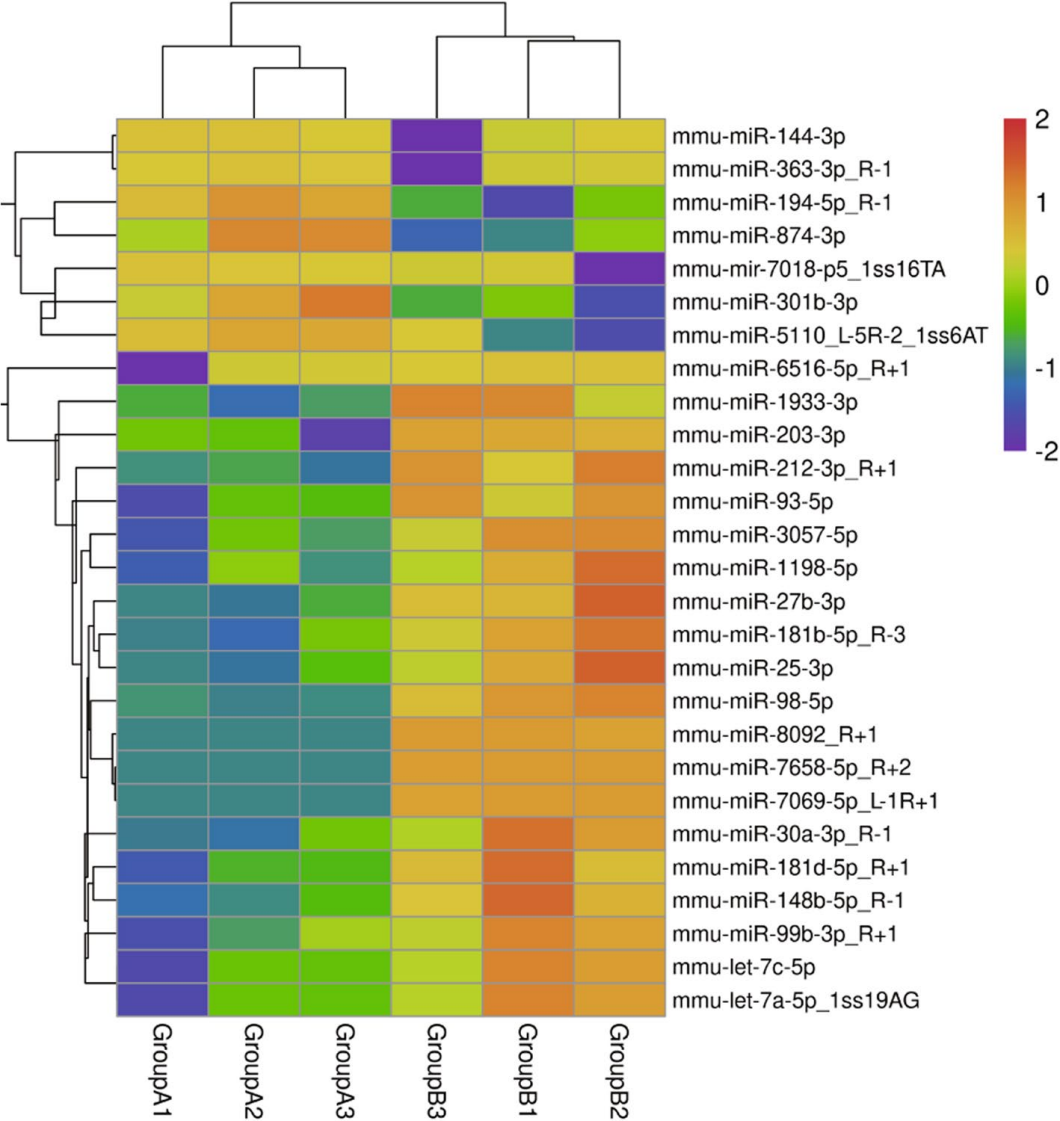
Heatmap of differently expressed miRNAs. The miRNA expression levels are presented as the log10-transformed normalized values. The different colours represent different miRNA expression levels. The colour range from blue to orange represents an expression level range from low to high; specifically, the dark blue colour indicates high expression, and the orange colour indicates low expression

**Fig. 5:**
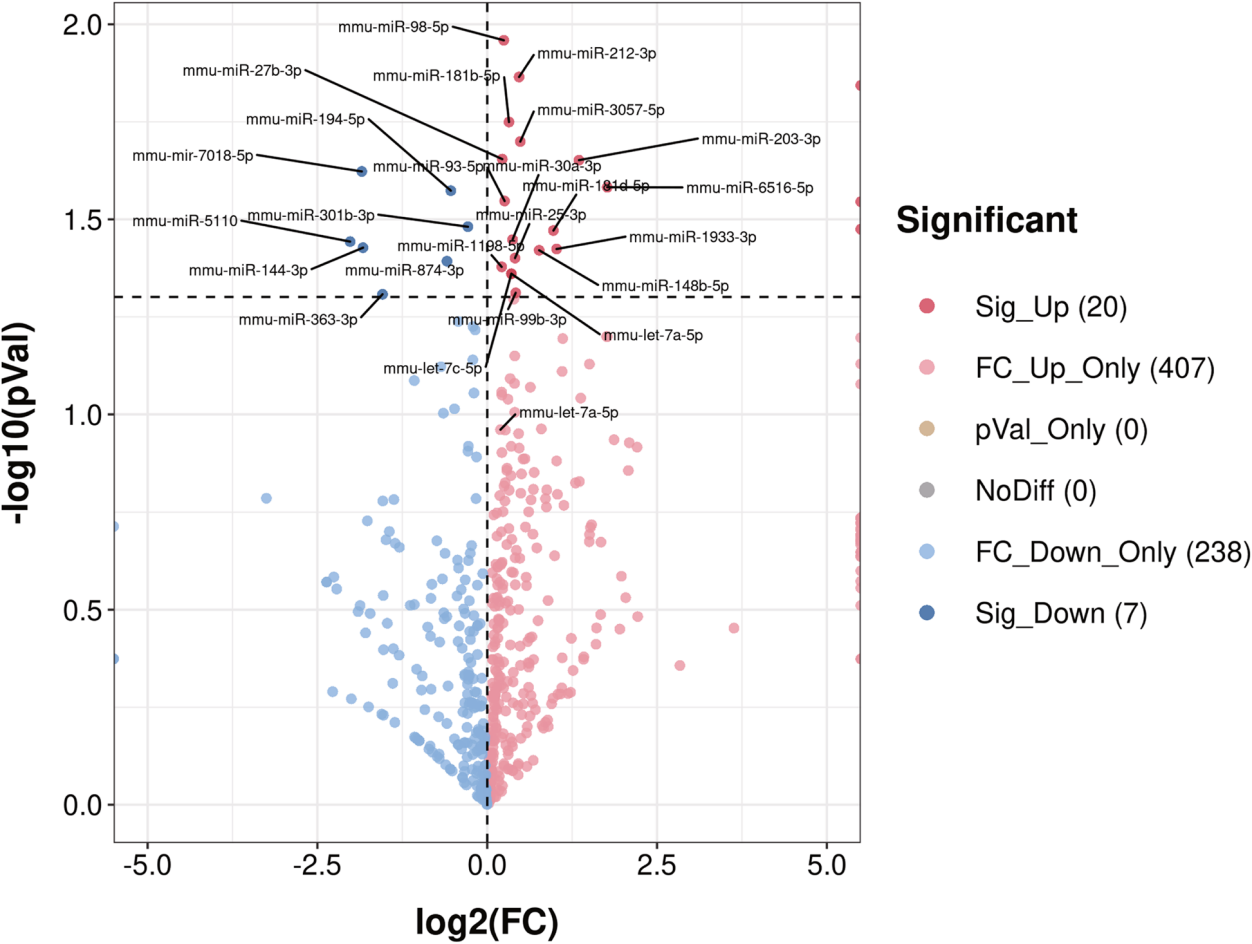
Volcano plot of differently expressed miRNAs. Log2(FC) represents the change in the differential expression of miRNAs in different samples; −log10(*p* value) represents the statistical significance of the difference in miRNA expression; the red colour indicates significantly differentially upregulated genes, the blue colour indicates significantly differentially downregulated genes, and the grey points represent genes showing nonsignificant differential expression

In total, 1853 miRNAs were detected, and 506 miRNAs overlapped between the infected group and non-infected group (Fig. 3). A differential miRNA cluster analysis intuitively displayed the specific expression profiles of the miRNAs in the different experimental treatments (Fig. 4), and the advanced volcano map displays the significant differential expression of multiple miRNAs (Fig. 5). The data showed that, compared with the non-infection group, there were 20 miRNAs upregulated and 7 miRNAs downregulated significantly in the infection group. The expression levels and the up- or downregulation relationships are presented in Table 2. Among the detected miRNAs, mmu-miR-27b-3p, − 93-5p, − 25-3p, − 1198-5p, −let-7c-5p and -let-7a-5p were expressed at relatively high levels. Target gene prediction and GO and KEGG enrichment analyses of these six significantly differentially expressed miRNAs were subsequently performed.

**Table 2:** Differentially expressed miRNAs and their expression levels

miRNA	Fold change	log2 FC	***p***-value	Exp level	Up/down
mmu-miR-27b-3p	1.17	0.22	2.22E-02	high	up
mmu-miR-93-5p	1.19	0.26	2.84E-02	high	up
mmu-miR-25-3p	1.33	0.41	3.98E-02	high	up
mmu-miR-1198-5p	1.16	0.21	4.19E-02	high	up
mmu-let-7c-5p	1.28	0.36	4.36E-02	high	up
mmu-let-7a-5p	1.28	0.36	4.36E-02	high	up
mmu-miR-7658-5p	inf	inf	1.44E-02	low	up
mmu-miR-7069-5p	inf	inf	2.85E-02	low	up
mmu-miR-8092	inf	inf	3.35E-02	low	up
mmu-miR-98-5p	1.18	0.24	1.10E-02	middle	up
mmu-miR-212-3p	1.38	0.47	1.36E-02	middle	up
mmu-miR-181b-5p	1.25	0.32	1.78E-02	middle	up
mmu-miR-3057-5p	1.40	0.49	2.00E-02	middle	up
mmu-miR-203-3p	2.55	1.35	2.23E-02	middle	up
mmu-miR-6516-5p	3.40	1.77	2.62E-02	middle	up
mmu-miR-181d-5p	1.97	0.97	3.38E-02	middle	up
mmu-miR-30a-3p	1.30	0.37	3.56E-02	middle	up
mmu-miR-1933-3p	2.03	1.02	3.77E-02	middle	up
mmu-miR-148b-5p	1.70	0.76	3.80E-02	middle	up
mmu-miR-99b-3p	1.34	0.42	4.88E-02	middle	up
mmu-mir-7018-p5	0.28	−1.85	2.38E-02	middle	down
mmu-miR-194-5p	0.69	−0.54	2.67E-02	middle	down
mmu-miR-301b-3p	0.82	−0.28	3.30E-02	middle	down
mmu-miR-5110	0.25	−2.02	3.61E-02	middle	down
mmu-miR-144-3p	0.28	−1.83	3.74E-02	middle	down
mmu-miR-874-3p	0.66	−0.59	4.05E-02	middle	down
mmu-miR-363-3p	0.34	−1.54	4.92E-02	middle	down

Finally, a number of 5643 target genes were predicted by using TargetScan (v5.0) and miRanda (v3.3a) software. GO and KEGG enrichment analysis of the screened target genes was then performed using the OmicStudio tools (https://www.omicstudio.cn/tool), and in total, 5576 target genes were effectively enriched.

### GO enrichment analysis

The biological process (BP) enrichment analysis revealed that 92.88, 71.98, 45.57, 43.33, 43.33, 34.36, 30.28, 27.73, 25.39 and 22.84% of the target genes mainly participated in signal transduction, the G protein-coupled receptor signalling pathway, response to stimulation, the detection of chemical stimulation related to olfactory perception, olfactory perception, the positive regulation of transcription through RNA polymerase II, transcription regulation of DNA template, multicellular biological development, cell differentiation and phosphorylation, respectively. In addition, 18.05% of the target genes also participated in the oxidation–reduction process, protein transport, phosphorylation and ubiquitination, ion transport, the cell cycle and the regulation of apoptosis.

As demonstrated by the cellular component (CC) enrichment analysis, 90.91, 63.42 and 58.69% of the target genes were involved in cell membrane composition, cytoplasmic composition, and plasma membrane composition, respectively. In addition, 52.03, 35.28, and 28.85% of the target genes participated in the composition of the nucleus, cytoplasmic cells, and nucleoplasm, respectively.

**Fig. 6:**
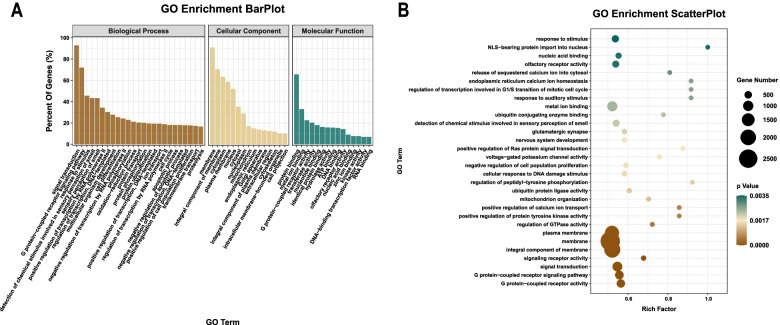
GO enrichment of the target genes. **A** Bar plot. **B** Scatterplot. The rich factor indicates the ratio of the number of differentially expressed genes divided by the total number of genes annotated with a specific term. For a term, a smaller *p*-value indicates a higher degree of enrichment. The diameter of the point represents the number of genes enriched with the item

The molecular function (MF) enrichment analysis demonstrated that 65.59, 33.03, 22.53 and 20.25% of the target genes participated in protein binding, metal ion binding, G protein-coupled receptor activity and transferase activity, respectively. In addition, some genes were found to be involved in the combination of nucleotides and ATP and DNA molecules. The results of the GO enrichment analysis are presented in Fig. 6.

### KEGG enrichment analysis

The results of the KEGG enrichment analysis revealed that the target genes primarily participated in the modulatory control of human diseases, metabolism and biological systems. In addition, a large proportion of genes were classified into the categories of cellular process, environmental information processing and genetic information processing. In this section, the percentage of genes refers to the percentage of significantly enriched genes in the corresponding secondary categories.

**Fig. 7:**
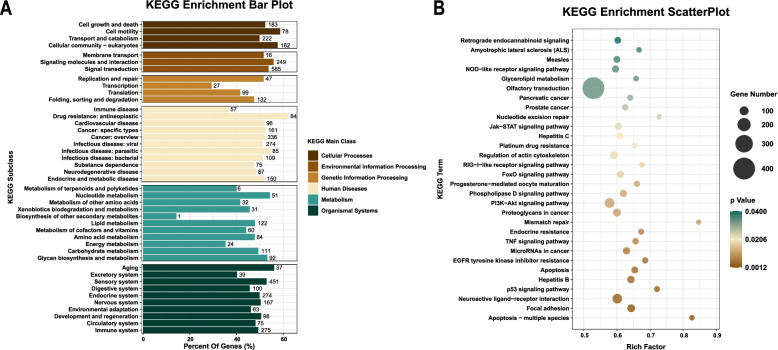
KEGG enrichment of the target genes. **A** Bar plot. **B** Scatterplot. The percentage of genes refers to the percentage of significantly enriched genes in the corresponding secondary categories

As shown in Fig. 7A, most significantly enriched target genes (almost three-quarters of all enriched genes) were related to infectious diseases (associated with viral, parasitic and bacterial infections) and cancer in the category of human diseases. In addition, the percentage of genes in each secondary category exceeded 50%. Within the metabolism category, 51 (54.26%), 122 (47.84%), 84 (47.73%), 111 (49.33%) and 92 (53.18%) target genes were significantly enriched in nucleotide metabolism, lipid metabolism, amino acid metabolism, carbohydrate metabolism, and glycan biosynthesis and metabolism, respectively. The percentage of enriched genes in each secondary category related to organismal systems exceeded 40% as follows: ageing (56.06%), sensory system (52.81%), development and regeneration system (50.52%), nervous system (50.3%), endocrine system (49.82%), environmental adaptation (46.11%), circulatory system (48.08%), immune system (49.28%), digestive system (45.66%), and excretory system (40.22%). Within the environmental information processing category, 249 genes were predominantly enriched in pathways related to signalling molecule interactions, 585 genes were related to signal transduction, and 16 genes were related to membrane transduction. Moreover, within the genetic information processing category, 47 genes were enriched in nucleotide replication and repair, and 132 genes were enriched in protein folding, sorting and degradation. Strikingly, the data related to the cell process category revealed that there were 183 target genes predominantly gathered in modulating the control of cell growth and death. Moreover, the rich factor analysis (Fig. 7B) showed that these target genes were predominantly enriched in apoptosis, focal adhesion, neuroactive ligand–receptor interaction, p53 signalling pathway and hepatitis B. This finding is crucial for our subsequent screening of the target genes involved in modulating apoptosis in macrophages infected with Mtb.

### Visualization analysis of the KEGG enrichment results

Metascape (https://metascape.org) was selected to visualize and analyse the biological functions of the target genes enriched in cell growth and death regulation. A total of 183 genes were transformed into their corresponding *M. musculus* Entrez gene IDs, and related enrichment analyses were then performed. The top 20 clusters and their representative enriched terms were converted into a network layout.

**Fig. 8:**
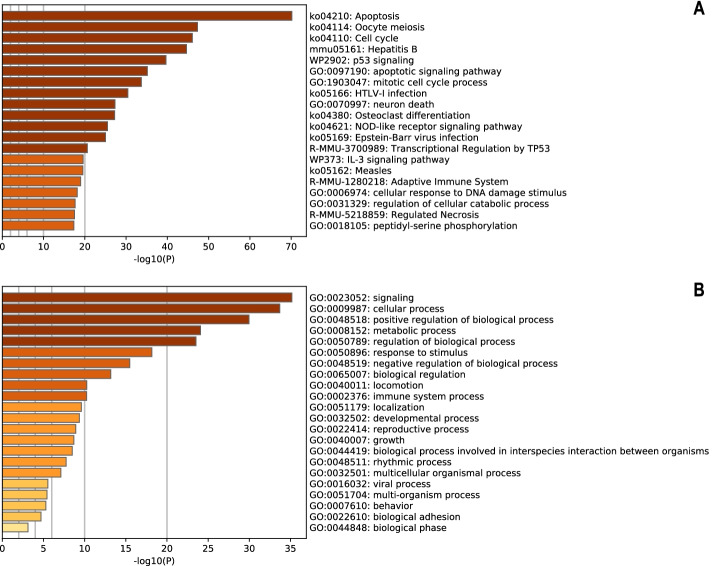
Bar graph of enriched terms across input gene lists (coloured by *p*-values). **A**. Top 20 enriched terms across input gene lists. **B**. Top Gene Ontology biological process terms

**Fig. 9:**
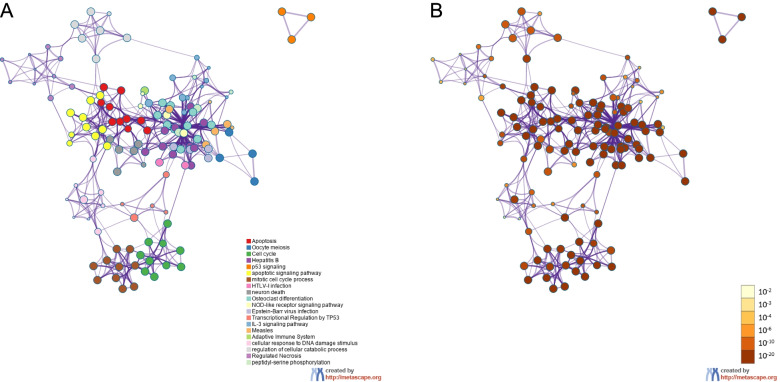
Network of enriched terms. **A** Coloured by cluster ID. Nodes that share the same cluster ID are typically close to each other. Terms with similarity scores > 0.3 are linked by edges (the thickness of the edges represents the similarity score). **B** Coloured by the *p*-values. Terms containing more genes tended to have more significant *p*-values. A darker colour indicates that the node is more statistically significant

As indicated in the bar graph shown in Fig. 8, apoptosis (ko04210), oocyte meiosis (ko04114), cell cycle (ko04110), hepatitis B (mmu05161) and p53 signalling (WP2902) were the top five significantly enriched terms across the input genes, and the specific gene annotation information of each cluster is summarized in Table 3. The result demonstrated that these genes were mainly involved in signalling, cellular processes and the regulation of metabolic and biological processes. The same result is displayed in Fig. 9, where each node stands for an enriched term, coloured first based on its cluster ID and then according to its *p* value.

**Table 3:** Twenty major groups showing enhanced enrichment

GO	Category	Description	Count	%	Log10 (P)	Log10(q)
ko04210	KEGG pathway	Apoptosis	51	27.87	−70.51	−66.23
ko04114	KEGG pathway	Oocyte meiosis	37	20.22	−47.57	−43.76
ko04110	KEGG pathway	Cell cycle	37	20.22	−46.32	−42.64
mmu05161	KEGG pathway	Hepatitis B	38	20.77	−44.52	−41.08
WP2902	WikiPathways	p53 signalling	28	15.30	−39.87	−36.63
GO:0097190	GO ***BP***	Apoptotic signalling pathway	52	28.42	−35.54	−32.46
GO:1903047	GO ***BP***	Mitotic cell cycle process	53	28.96	−34.08	−31.02
ko05166	KEGG pathway	HTLV-I infection	36	19.67	−30.68	−27.83
GO:0070997	GO ***BP***	Neuron death	39	21.31	−27.54	− 24.82
ko04380	KEGG pathway	Osteoclast differentiation	26	14.21	−27.41	−24.69
ko04621	KEGG pathway	NOD-like receptor signalling pathway	27	14.75	−25.68	−23.10
ko05169	KEGG pathway	Epstein–Barr virus infection	29	15.85	−25.22	−22.66
R-MMU-3700989	Reactome ***GS***	Transcriptional regulation by TP53	28	15.30	−20.80	−18.48
WP373	WikiPathways	IL-3 signalling pathway	19	10.38	−19.72	−17.46
ko05162	KEGG pathway	Measles	21	11.48	−19.68	−17.43
R-MMU-1280218	Reactome ***GS***	Adaptive immune system	37	20.22	−19.23	−17.01
GO:0006974	GO ***BP***	Cellular response to DNA damage stimulus	39	21.31	−18.33	−16.14
GO:0031329	GO ***BP***	Regulation of cellular catabolic process	38	20.77	−17.85	−15.69
R-MMU-5218859	Reactome ***GS***	Regulated necrosis	14	7.65	−17.59	−15.44
GO:0018105	GO ***BP***	Peptidyl-serine phosphorylation	27	14.75	−17.53	−15.40

“Count” is the number of genes in the lists annotated with a given ontology term. “%” is the percentage of all genes annotated with a given ontology term (only input genes annotated with at least one ontology term were included in the calculation). “Log10(P)” is the log base 10-transformed *p*-value. “Log10(q)” is the log base 10-transformed multitest adjusted *p*-value. ***BP****:* Biological process; ***GS****:* Gene sets

**Fig. 10:**
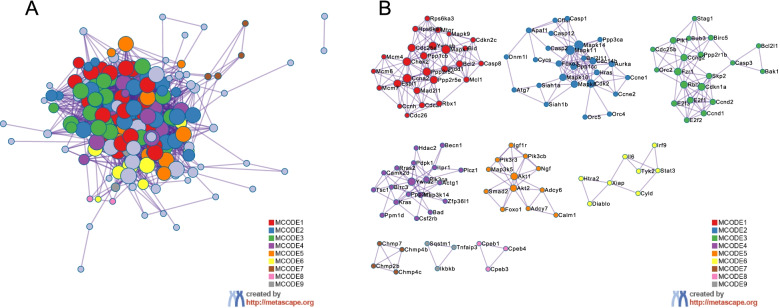
Protein–protein interaction enrichment analysis. Protein–protein interaction network and MCODE components identified in the gene lists. The MCODE algorithm was applied to the network to identify the neighbourhoods of protein-intensive connections. Each MCODE network is assigned a unique colour. MCODE: Molecular Complex Detection

An extended protein–protein interaction network was framed, and 9 clusters were extracted using the MCODE algorithm. The pathways and biological processes enrichment analysis was utilized to each MCODE component independently, as shown in Fig. 10. Three optimal scoring terms were preserved as the functional description of the relevant components and highlighted in Table 4. The results demonstrated that the enriched genes were predominantly involved in regulating the cellular apoptosis process and oocyte meiosis. MCODE_1 and MCODE_3 were associated with the regulation of the cell cycle; MCODE_2 and MCODE_4 mostly participated in apoptosis pathways; MCODE_5 was associated with the longevity regulating pathway; and MCODE_6 was associated with an interleukin-6 family signalling pathway and CD4^+^ or CD8^+^ α-β T cell lineage commitment. As shown by the data presented in Table 5, apoptosis was the term with the best score obtained from the MCODE analysis.

**Table 4:**
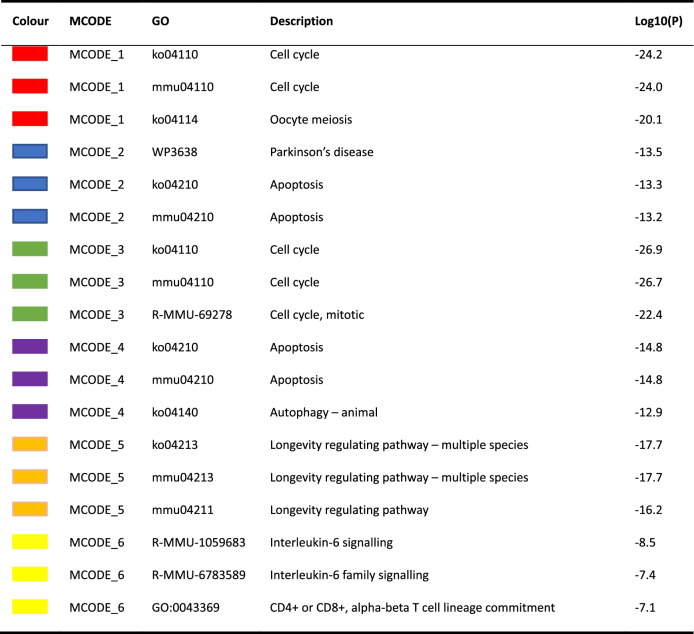
Six MCODE component annotation details

If the network contained between 3 and 500 proteins, the Molecular Complex Detection (MCODE) algorithm was applied to identify densely connected network components

**Table 5:** Top three MCODE terms

GO	Description	Log10(P)
ko04210	Apoptosis	−71.5
mmu04210	Apoptosis	−70.9
ko04114	Oocyte meiosis	−48.3

A GO enrichment analysis of each MCODE network was performed to assign “meanings” to the network components, and only the top three terms based on the *p*-value were retained

### The results of exosomal miRNA validation by qRT-PCR

**Fig. 11:**
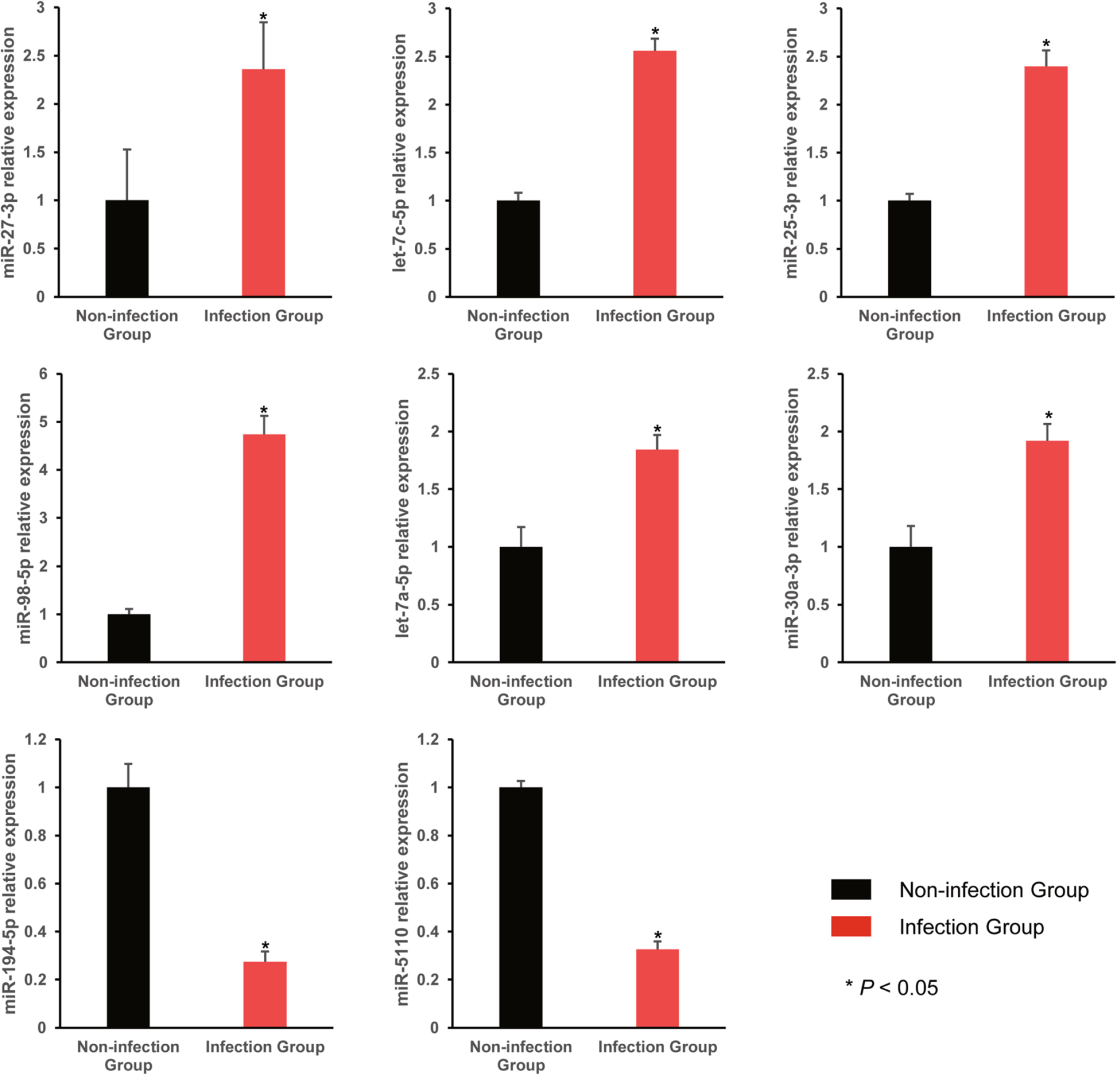
Validation of relative miRNA expression in exosomes by qRT–PCR. A1-A3: Non-infection group. B1-B3: Infection group

Finally, eight miRNAs including mmu-miR-27-3p, mmu-let-7a-5p, mmu-let-7c-5p, mmu-miR-25-3p, mmu-miR-98-5p, mmu-miR-30a-3p, mmu-miR-194-5p and mmu-miR-5110 were randomly selected from the list of differentially expressed miRNAs, and their relative expression levels in each group were detected and presented in Fig. 11. Compared with the non-infected group, the relative expression of mmu-miR-27-3p, mmu-let-7a-5p, mmu-let-7c-5p, mmu-miR-25-3p, mmu-miR-98-5p and mmu-miR-30a-3p in the infection group were up-regulated, and the relative expression of mmu-miR-194-5p and mmu-miR-5110 were decreased in the infection group. The difference between the two groups was statistically significant (*p* < 0.05). Their expression tendencies were consistent with the sequencing data. It is indicated that the result of high-throughput sequencing was accurate and reliable.

## Discussion

In the present study, six miRNAs differentially expressed in exosomes derived from RAW264.7 cells infected with BCG were preliminarily screened. The bioinformatics and enrichment analysis demonstrated that these exosomal miRNAs and their target genes were predominantly involved in multiple biological processes and signal pathways related to cellular growth and death.

TB remains a fatal threat to global human health and safety and ranks first in terms of mortality rate among all infectious diseases, and no optimal treatment for the effective control of TB infection has been established. Mtb is a typical intracellular pathogen that causes TB infection and mainly infects macrophages, which serve as the initial sites of pathogen survival and replication; and this finding also provides a potential mechanism for controlling the activation of uninfected macrophages mediated by T cells [18]. Once a pathogen is phagocytosed by macrophages, the body activates the immune defence system to eliminate the pathogen. In contrast, to adapt to its new environment, Mycobacteria competitively manipulate the network of host cell miRNAs to facilitate their survival during the host-pathogen interaction process. Any change in the structure or expression level of miRNAs may seriously affect biological processes and lead to the occurrence of pathological conditions [19].

In contrast, Mycobacterium infection can also alter the host cell exosome content. Our high-throughput sequencing data provided the best evidence. As shown in Fig. 3, a total of 1853 miRNAs were detected from the final valid data filtered from the raw data; among them, 580 in the non-infected group and 602 in the infection group; 506 were overlapped between the two groups. There were 74 unique miRNAs in the non-infected group and 96 in the infection group. The number of exosomal miRNA in the infection group was more than 22 in the non-infected group. The results indicated that Mycobacteria could obviously change the macrophage exosomes contents such as miRNA.

Macrophages constitute the front line of defence in innate immunity, and apoptosis plays a critical role in the clearance of intracellular pathogenic bacteria [20]. However, Mycobacteria have the ability to hijack the metabolism and energy pathways of host cells and disrupt the autophagy and apoptosis processes of infected cells [21]. An increasing number of studies have found that Mtb is capable of evading immune surveillance and promoting its retention and survival in macrophages by manipulating the host cell miRNA network [22–24]. Some of the let-7 family members were reported that they play an important role in the immune response to TB infection [25, 26]. Several accurately expressed miRNAs, such as hsa-miR-144-3p, − 142-3p and -23a-5p, have also been shown to be strongly associated with the immune response to TB infection. Moreover, miR-142-3p can suppress phagocytosis by perturbing neural Wiskott-Aldrich protein syndrome in host cells [27]. By targeting ATG4a, miR-144-3p can prevent the autophagy process activation and promote bacillus infection and survival in BCG infected RAW264.7 cells [28]. In the course of Mtb infection, miR-23a-5p may influence the TLR2/MYD88/NF-κB pathway by targeting TLR2 to manipulate autophagy and Mtb persistence [29].

Thus far, most previous studies have attempted to reveal the complex mechanisms of host-pathogen interactions at the cellular miRNA level, but the detailed roles of miRNAs in TB infection remain poorly understood. Therefore, we explored the possible related mechanisms at the exosomal miRNA level and attempted to unravel the secretion of Mtb during immune evasion. Our study demonstrated that the exosomal miRNAs expression level from the cell culture medium of macrophages between the two groups showed significant differences. Twenty-seven differentially expressed miRNAs were screened from the comparison of the infection and non-infection group, and these included 20 upregulated and 7 downregulated miRNAs, respectively, in the infection group. Among them, mmu-miR-27b-3p, − 93-5p, − 25-3p, − 1198-5p, −let-7c-5p and -let-7a-5p were the top six significantly differentially upregulated miRNAs (*p* < 0.05) and fit for the subsequent analysis.

It was demonstrated that majority of the target gene products were located in the membrane, cytoplasm, plasma membrane, nucleus, cytosol, nucleoplasm and mitochondrion; their molecular functions were mostly enriched in protein and metal ion binding, G protein-coupled receptor activity, protein and metal ion binding and transferase activity; furthermore, the target genes were involved in multiple biological processes, including the G protein-coupled receptor signalling pathway, signal transduction, the response to stimulation, the detection of chemical stimulation related to olfactory perception and the positive modulation of transcription through RNA polymerase II.

The KEGG enrichment analysis demonstrated that the predicted target genes widely participated in the regulation of pathways related to the following categories, including human diseases, metabolism, cellular processes, environmental information processing, genetic information processing and organismal systems. The results of this analysis contains apoptosis, focal adhesion, neuroactive ligand–receptor interaction, the p53 signalling pathway, hepatitis B, EGFR tyrosine kinase inhibitor resistance, microRNAs in cancer, the TNF signalling pathway, endocrine resistance, mismatch repair and the PI3K-Akt signalling pathway (*p*<0.05).

Mtb is a slow-growing facultative intracellular parasite. Macrophages are not only the first stop during the host invasion process of Mtb but also serve as a potential niche used by mycobacteria to form latent infections. The prognosis of macrophages infected with Mtb determines the fate of intracellular pathogens. We further focused our attention on the target genes which closely connected to the modulation of cell growth and death pathways. In total, 183 target genes were collected for further data mining and functional visualization analysis using the Metascape online tool. The analysis of the PPI network and MCODE displayed that these proteins were tightly related to the cell cycle, oocyte meiosis, Parkinson’s disease, apoptosis, the longevity regulating pathway and interleukin-6 signalling. It is apparent that apoptosis was the predominant term.

Similar findings were reported [30], Aplipoor et al. infected human monocyte-derived macrophages with BCG and identified a complex set of exosomal miRNAs. The enrichment analysis illustrated that these miRNAs were primarily taken part in the regulation of pathways related to host metabolic progression, cell signalling and infectious diseases. The result suggested that metabolic pathways participating in protective immunity of the host are weakened to favour bacterial survival within macrophages, and this effect ultimately leads to the persistence of intracellular bacteria. In another study, the researchers further investigated three miRNAs (miR-1224, − 484, and − 425) that regulate metabolic pathways during TB infection [31]. They found that the increase in the relative expression level of miR-1224 was higher than miR-484 and miR-425 expression in the BCG-infected group. They hypothesized that Mtb was capable of tolerating the hostile microenvironment by triggering and subverting (repatterning) host metabolic pathways, and as a result, Mtb escapes degradation by lysosomes and forms a lipid-rich niche that favours its survival. Previous studies have confirmed that the modification of lipid metabolism in host cells plays a key role in intracellular bacterial retention during TB infection [32, 33]. MiR-1224 also participates in the modulation of lipid metabolism.

As a type of programmed death, apoptosis is one of the important mechanisms by which macrophages govern TB infection in the host innate immune defence. However, the mechanism through which Mycobacteria trigger macrophage apoptosis remains largely unknown. Some studies have partially uncovered the regulatory mechanisms underlying the interplay between miRNAs and the host macrophage immune response and apoptosis during TB infection [22]. A microarray profiling survey revealed that the expression of miRNAs derived from CD14^+^ monocytes of active pulmonary TB patients was downregulated, and nothing but miR-20a-5p expression could be conversed after successful anti-tuberculosis therapy [34]. Functional in vitro experiments investigating anti-tuberculosis infection manifested that the downregulation of miR-20a-5p could trigger apoptosis in macrophages by targeting JNK2 to eliminate intracellular Mycobacterium. In another study, it was showed that the overexpression of miR-20b-5p in Mtb-infected macrophages was able to reduce the cell viability and induce apoptosis; conversely, the inhibition of the expression of miR-20b-5p promoted cell viability and weakened macrophage apoptosis. Mcl-1 was one of the predicted targets of miR-20b-5p, and the downregulation of this miRNA could upregulate Mcl-1 expression to facilitate the intracellular survival of Mycobacteria [35].

Above all, we found 27 differentially expressed exosomal miRNAs in exosomes derived from BCG infected RAW264.7 cells, their target genes were positively corelated with the regulation of apoptosis. This discovery provides relatively strong evidence revealing the mechanism through which Mycobacteria evade the immune system at the exosomal miRNA level. Furthermore, it also provides an important direction for the subsequent screening of specific miRNAs to investigate the detailed mechanism of host-pathogen interactions during TB infection for our team. Eight miRNAs, namely, mmu-miR-27b-3p, − 25-3p, − 98-5p, −30a-3p, − 194-5p, − 5110, −let-7a-5p and -let-7c-5p, were randomly selected from the list of differentially expressed miRNAs to verify the accuracy of the high-throughput sequencing data by qRT–PCR. The relative expression of mmu-mir-27b-3p, −let-7c-5p, − 25-3p, − 98-5p, −let-7a-5p and -30a-3p in the infection group indicated a consistent trend with the previous sequencing results, but the relative expression levels of the rest two miRNAs were contrary to the results obtained by sequencing, which may be related to the two different methods used for the exosome isolation and exosomal RNA extraction. Overall, the relative expression levels of the miRNAs showed significant differences between the infection and non-infection group (*p* < 0.05), which indicated that the high-throughput sequencing results were relatively accurate.

Exosomes are an important area of research related to human diseases and infectious agents that has sparked interest in recent years. Because exosomes have the characteristics of biocarriers and possess information regarding the body’s pathological state with respect to the immune system, they may be used as regulators of the immune response to activate the regulation of the host immune response and infection [36]. In our study, high-throughput sequencing revealed that the miRNA expression profiles changed in exosomes derived from macrophages infected with BCG. The discovery of these differentially expressed miRNAs is conducive to our recognition of the complicated mechanism of host-pathogen interactions during the pathogenesis of TB at the exosomal miRNA level. In addition, using specimens from patients with clinically confirmed TB and healthy volunteers in the sequencing analysis and validation of the results would be more clinically relevant and rigorous.

## Conclusion

It is concluded that Mycobacteria could alter the expression profile of miRNA in exosomes derived from macrophage after *Mycobacterium Bovis* Bacillus Calmette-Guérin infection, and the differentially expressed miRNAs were involved in multiple biological processes and signalling pathways. The top six up-regulated miRNAs and their targeted genes were predominantly correlated with the regulation of apoptosis. However, the mechanisms through which Mycobacteria evades immune surveillance during its infection remain complex. The interplay between pathogens and host cells is an intricately linked event involving multiple factors, such as the Mycobacteria species, the bacteria virulence, the duration of the infection stimulus, even the status of macrophages and other immune cells, which might influence the prognosis and outcomes of TB.

## Data Availability

The datasets generated during the current study are available in the Genome Sequence Archive (Genomics, Proteomics & Bioinformatics 2021) in National Genomics Data Center (Nucleic Acids Res 2021), China National Center for Bioinformation / Beijing Institute of Genomics, Chinese Academy of Sciences (GSA: CRA006010) that are publicly accessible at https://ngdc.cncb.ac.cn/gsa/s/ITsJl588.

## Abbreviations

TB: Tuberculosis
BCG: Bacillus Calmette-Guérin
MTBC: Mycobacterium tuberculosis complex
Mtb: Mycobacterium tuberculosis
DMEM: Dulbecco’s modified Eagle’s medium
FBS: Foetal bovine serum
PBS: Phosphate buffered saline
GO: Gene Ontology
KEGG: Kyoto Encyclopedia of Genes and Genomes
MCODE: Molecular Complex Detection
BLAST: The Basic Local Alignment Search Tool
